# SCANellome: Analysis of the Genomic Diversity of Human and Non-Human Primate Anelloviruses from Metagenomics Data

**DOI:** 10.3390/v15071575

**Published:** 2023-07-19

**Authors:** Florian Laubscher, Laurent Kaiser, Samuel Cordey

**Affiliations:** 1 Laboratory of Virology, Department of Diagnostics, Geneva University Hospitals & Faculty of Medicine, University of Geneva, 1205 Geneva, Switzerland; florian.laubscher@hcuge.ch (F.L.); laurent.kaiser@hcuge.ch (L.K.); 2 Division of Infectious Diseases, Geneva University Hospitals, 1205 Geneva, Switzerland; 3 Geneva Centre for Emerging Viral Diseases, Geneva University Hospitals, 1205 Geneva, Switzerland

**Keywords:** SCANellome, metagenomics, anelloviruses, genomic diversity

## Abstract

Anelloviruses are extremely prevalent in the human population and are considered to be commensal parts of the human virome. The best-known member in humans is the Torque teno virus. Recent metagenomic next-generation sequencing investigations have helped reveal the considerable number of species and genotypes from the same genus that can be co-detected within a single individual and that this diversity increases as a function of age during the first months/years of life. As a result, to date, the bioinformatics analysis of this genetic diversity remains complex and constraining for researchers. Here, we present SCANellome, a user-friendly tool to investigate the anellome composition at the genus, species, and genotype levels of samples from metagenomics data generated by the Illumina and Nanopore platforms. SCANellome is based on an in-house up-to-date database that includes all human and non-human primate anellovirus reference sequences available on GenBank and meets the latest classification criteria established by the International Committee on Taxonomy of Viruses.

## 1. Introduction

Anelloviruses are non-enveloped viruses, containing a circular, negative-sense, single-stranded DNA genome. According to the recent taxonomic update by the International Committee on Taxonomy of Viruses (ICTV), the family Anelloviridae comprises 30 genera [1]. Four of them have been reported in humans: Alphatorquevirus, Betatorquevirus, Gammatorquevirus, and Hetorquevirus, more commonly called Torque teno virus (TTV), Torque teno mini virus (TTMV), and Torque teno midi virus (TTMDV), respectively, for the first three [2,3,4]. The current consensus is that Alpha-, Beta-, and Gammatorquevirus are mostly acquired early in life in humans [5,6,7] and are considered as commensal parts of the human virome [8]. TTV is to date the most studied human anellovirus since it has been suggested to represent a potential biomarker of immunity (i.e., the TTV viral load in blood correlates with the level of host immunosuppression) and to help predict some clinical outcomes in transplant recipients [9,10,11,12,13].

It is known (and seems to be the rule) that a single individual (a child or adult) can be co-infected by a large number of different human anellovirus species and genotypes of the same genus [14,15,16,17]. We recently reported a significant trend toward an increase in TTV genomic diversity with age in a cohort of children under five years of age [17].

Does this trend continue during the rest of childhood, and how long is such diversity maintained? Does the increase in TTV blood viral load reported in the case of immunosuppression reflect an increase in all species/genotypes present initially or do only some become predominant? Are some genotypes more frequently associated with certain specific clinical manifestations? Are the designs of the primers and probes used in the real-time PCR used to qualitatively or quantitatively detect TTV, TTMV, or TTMDV optimal for the detection of all of their respective genotypes? Here are just some examples of questions that only detailed analyses of genomic diversity could be able to answer. Therefore, it is essential that human virome investigations based on metagenomics next-generation sequencing (mNGS) methods no longer solely report the presence of “TTV”, “TTMV”, or “TTMDV” as is still frequently the case. They should also analyze and report the complete genomic diversity of human anellovirus genera, species, or genotypes, improving our knowledge of the intra-host “human anellome” dynamic (e.g., longitudinal studies), as well as providing a better overview of the genomic diversity in various parts of the world. However, an in-depth analysis of human anellovirus genomic diversity from raw data requires not only substantial bioinformatics work depending on the number of samples to be analyzed but also ensuring that the database is up-to-date and meets the latest classification criteria established by the ICTV.

Here, we introduce the “SCANellome” software that we developed to analyze raw data generated from the Illumina or Nanopore platforms and report the genomic diversity of anelloviruses from metagenomics data at the genus, species, and/or genotype levels. If needed, consensus sequences can be generated. This software is based on an automated database that includes all primate (both human and non-human) anellovirus reference sequences available on GenBank classified according to the latest classification criteria established by the ICTV. Furthermore, the software is designed so that if new classification criteria are released by the ICTV, they can be easily integrated into the regular updates.

## 2. Materials and Methods

### 2.1. Primate Anelloviruses Database

**Figure 1:**
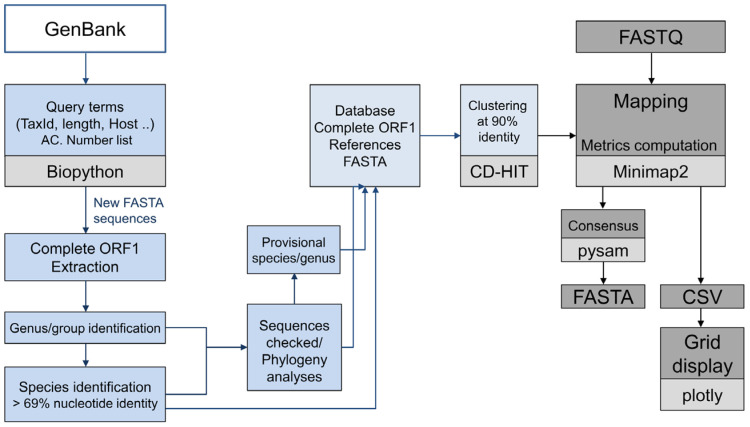
SCANellome flowchart. The blue boxes correspond to the different steps carried out to generate the complete ORF1 primate anelloviruses database. The light grey boxes are the software packages. The dark gray boxes correspond to the SCANellome input and output steps.

The complete ORF1 primate anellovirus database is an in-house FASTA database based on GenBank sequences (Figure 1). The database is annotated at the genus and species levels. The database is maintained using Bash and Python script.

The current version used in SCANellome was updated on 19 April 2023. After each update, new primate anellovirus sequences are downloaded using combinations of query terms (sequence length, organism txid, and host) in Biopython (version 1.81) [18] requests to GenBank. Accession numbers of sequences already processed are stored in a lookup table. The ORF1 sequence is extracted, and completeness is assessed (looking at the six possible reading frames and including alternative start codons for TTV group 4). For the convenience of classification and to speed up the comparison process, the TTV, TTMV, and TTMDV species were divided into groups. Historical groups were used for TTV (1, 2, 3, 3a, 3b, 3c, 4, 5, 6, and 7) only. Although the notion of groups does not represent an official classification criterion, publications frequently refer to it for TTV. Therefore, SCANellome further provides (indicative only) the presence of groups. Based on the phylogenetic analyses, we split TTMV and TTMDV into ten (A, B, C, D, E, F, G, H, J, and K) and five groups (A to E), respectively. Within the genera or groups, species identification is made by comparing the sequence identities one by one, aligned using MUSCLE (version 3.7). Sequences that do not fit the species assignment criteria are reviewed. If unassigned sequences do not show problems (e.g., an incomplete ORF) or are not recombinant sequences, provisional new species are assigned. Up to the current database version, all divergent new species that did not belong to the three classical human genera were further phylogenetically analyzed, and the species were either assigned to the Hetorquevirus genera or to four novel genera named Lamedtorquevirus, Memtorquevirus, Samektorquevirus, and Yodtorquevirus (following ICTV naming recommendations). These novel genera and species have been proposed to the Anelloviridae ICTV committee (at the time of the manuscript review process, our proposal was reviewed by the whole Anelloviridae study group who supported and submitted it to the ICTV proposal secretary). The TTMV and TTMDV provisional species are temporarily named using an “Unclassified-” prefix and alphanumerical suffix depending on the groups (e.g., Unclassified-TTMV-001A). Currently, the database contains 142 and 50 provisional TTMV and TTMDV species, respectively.

The total ORF1 database is composed of over 17K sequences, divided into 11 genera (Table 1). Using CD-HIT at 90% identity, a representative FASTA database was generated to be used as a reference database in SCANellome. Of note, because of the constant discovery of new anellovirus sequences, potential new TTMV and TTMDV species have not yet been formally proposed as novel species to avoid the creation of species that will not be maintained because of the possible lack of sequences that share nucleotide identity with potential newly identified species.

### 2.2. SCANellome

SCANellome is implemented entirely in Python 3. The Tkinter module is used for the graphical interface [19], the mappy 2.24 python module of minimap2 is used for the mapping of the reads [20], the pysam module (https://github.com/pysam-developers/pysam, accessed on 21 April 2023) is used for consensus sequence generation [21,22,23], and the plotly module is used for graphical data representation (HTML) [24]. SCANellome software is provided as a standalone executable. SCANellome is available for Linux (tested on Ubuntu versions 18.04.6 LTS and 20.04.6 LTS) and macOS (tested on version Ventura 13.3.1).

SCANellome aims to analyze Fastq files, which are selected for analysis via the graphical interface and offers the ability to create and save several projects. Samples are added to projects using the file name prefixes of the Fastq files. The results are stored in CSV format in the software and additional samples can be later added to any project. The project can be deleted as well. Inside a project, the analyzed samples can be selected to be graphically displayed as a heatmap sorted by anellovirus genera on an HTML page.

For the analysis, the following options are available: Illumina single-end, Illumina paired-end, and Oxford Nanopore, depending on the method used to obtain the Fastq. Fastq files have to be demultiplexed and can be additionally pre-processed. During the analysis, reads are mapped using mappy 2.24 to our completed ORF1 nucleotide sequences of the primate anelloviruses database (Figure 1). The Mappy “map-ont” preset is used if Oxford Nanopore is selected. Metrics are computed for all mapped references. For each anellovirus species, the results for the sequence reference with the best ORF1 coverage are selected. The presence of the species is reported if at least 50 percent of the complete ORF1 sequence is covered by reads.

When the analysis is completed, the results can be saved in a CSV file with the following fields:
1. Sample Name<name of the sample retrieved from the fastq name>2. ACC. NUMBER<GenBank accession number of the matching reference sequence>3. Reads<number of reads>4. ref_len<length of the ORF1 of the matching reference sequence>5. cov<length of the matching reference sequence covered by the reads>6. %cov<percentage of the matching reference sequence covered by the reads>7. depth<median depth>8. GENUS<genus of the reference sequence>9. GROUP<group within the genus>10. SPECIES<species of the reference sequence>11. GENOTYPE<genotype of the reference sequences if any>12. HOST<host infected by the reference sequence>

Optionally, consensus sequences can be generated using Pysam (version 0.19.1; default parameters).

The ORF1 consensus sequences of all reported anelloviruses can be saved as one FASTA file. The completeness and quality of the consensus can depend on the number of mapped reads, the Fastq quality, and pre-processing of the Fastq file. This step is slower and needs more temporary disk space because temporary SAM files are written.

To ensure specificity and sensitivity, 16 test datasets were generated in silico, each with 33 sequences ranging from 100% to 85% nucleotide sequence identity, with sequences from our database. For each genus or group, a sequence was randomly selected from the database (a total of 33), diversity was artificially added to the complete genome nucleotide sequence, and Fastq files were generated using art_illumina (version 2.5.8) (parameters: -ss HS25 -f 1000 -p -l 100 -m 250 -na -s 15). Above 92% identity, all viruses were detected; below 88% identity, no viruses were detected anymore. Overall, there were no false positives (Table S1). Furthermore, SCANellome was evaluated on 10 blood samples of which we had previously analyzed and published the genomic diversity of TTV [17]. This comparative analysis shows the robustness of SCANellome, which successfully detected all of the TTV species reported in our previous study. In addition, SCANellome highlights additional TTV species and correctly reclassifies species that have since been merged (Table S2). However, SCANellome has some limitations: the software does not generate complete viral sequences (SCANellome is restricted to the ORF1 database) nor does it report potential recombination events.

## 3. Conclusions

SCANellome represents an easy-to-use tool to investigate the anellome composition of samples from metagenomics data based on an up-to-date database that meets the latest classification criteria established by the ICTV. Thanks to SCANellome, such an analysis becomes accessible for any researchers in the anellovirus or more broadly in the human or primate virome fields.

## Figures and Tables

**Table 1:** Summary of the number of sequences and species for each genus in the complete ORF1 primate anelloviruses database.

Genus	Number of Complete ORF1 Sequences	Representatives 90% Identity	Number of Species
Alphatorquevirus	5444	419	36
Betatorquevirus	6659	1856	178
Gammatorquevirus	5534	1376	64
Hetorquevirus	109	15	7
Yodtorquevirus	17	2	2
Lamedtorquevirus	33	5	1
Memtorquevirus	55	20	3
Samektorquevirus	124	32	4
Omegatorquevirus	3	3	3
Zetatorquevirus	1	1	1
Epsilontorquevirus	1	1	1
Total	17,980	3730	300

## Data Availability

SCANellome is freely available at: https://laubscher.github.io/Anelloviruses/SCANellome.html (release date 3 July 2023), the source code is available at: https://github.com/Laubscher/SCANellome (release date 4 July 2023), the dataset is available at: https://doi.org/10.5281/zenodo.7937276 (release date 15 May 2023), and the complete ORF1 primate anelloviruses database is available at: https://github.com/Laubscher/Anelloviruses/releases/tag/Anellovirus_2023.1 (release date 27 April 2023).

## Supplementary Materials

The following supporting information can be downloaded at: https://www.mdpi.com/article/10.3390/v15071575/s1. Table S1: Dataset results; Table S2: Comparative analysis of the genomic diversity of TTV in 10 blood samples between SCANellome and the results previously obtained with a 2021 database. Abolished species are highlighted in blue (Torque teno virus 8 merged to Torque teno virus 7; Torque teno virus 11 merged Torque teno virus 9; Torque teno virus 12 merged Torque teno virus 9; Torque teno virus 22 merged to Torque teno virus 24; Torque teno virus 28 merged to Torque teno virus 29; Torque teno virus 39 merged to Torque teno virus 7; Torque teno virus 44 merged to Torque teno virus 20; and Torque teno virus 46 merged to Torque teno virus 21/24).
